# Latitudinal Variation in Estuarine Archaeal Biogeography: Deterministic vs. Stochastic Assembly Processes and Network Stability Across China’s Coastal Ecosystems

**DOI:** 10.3390/microorganisms14040752

**Published:** 2026-03-27

**Authors:** Yingpai Liu, Guoqing Lv, Zeyu Zhang, Ziyan Fu, Guo Yuan, Jiale Ding, Shuhan Wang, Yingjie Ma, Yaqi Song, Xiaoshuang Zhao, Mao Ye, Yonghui Wang, Zongxiao Zhang

**Affiliations:** 1 College of Geography Science and Tourism, Xinjiang Normal University, Urumqi 830054, China; 2 Xinjiang Laboratory of Lake Environment and Resources in Arid Zone, Xinjiang Normal University, Urumqi 830054, China; 3 Technical Research Center for Environmental Geotechnical Engineering Restoration and Resource Utilization, Xinjiang Normal University, Urumqi 830054, China

**Keywords:** estuarine archaeal communities, latitudinal biogeography, assembly processes, ecological networks, environmental filtering

## Abstract

Latitudinal gradients are widely recognized as a key macro-environmental driver shaping microbial biogeographic patterns; however, the spatial organization of sediment archaeal communities in estuarine ecosystems and the mechanisms underlying their assembly remain insufficiently understood. This study is based on sediment samples collected from three representative estuarine regions spanning distinct latitudes along the Chinese coastline—the North China Sea (NCS), East China Sea (ECS), and South China Sea (SCS). Based on 16S rRNA high-throughput sequencing, combined with null-model inference and molecular ecological network (MEN) analyses, we characterized latitudinal patterns in archaeal community distributions, assembly processes, and cross-regional interaction architectures. The results showed that archaeal communities exhibited obvious spatial segregation across three regions, with both community richness and network complexity increasing significantly toward lower latitudes. Nitrate (NO_3_^−^), ferric iron (Fe^3+^), and dissolved oxygen (DO) were identified as key environmental factors governing archaeal community structure. Notably, archaeal community assembly processes exhibited a clear latitudinal gradient: deterministic processes, particularly environmental filtering, were more obvious at lower latitudes, whereas the contributions of stochastic processes—including dispersal limitation and ecological drift—increased markedly at higher latitudes. A MEN analysis further revealed that archaeal networks at lower latitudes exhibited higher connectivity, modularity, and stability, suggesting that interspecific interactions may enhance ecosystem resistance to environmental disturbance under more stable environmental conditions. Overall, this study demonstrates that macro-environmental gradients jointly shape archaeal biogeographic patterns via multiple pathways, including modulation of environmental filtering, dispersal dynamics, and cross-regional interactions. These findings deepened our understanding of the stable mechanisms governing the diversity and biogeographical distribution of archaea in estuarine systems.

## 1. Introduction

Estuarine ecosystems, as transitional interfaces between terrestrial and marine realms, play a pivotal role in global biogeochemical cycling [1]. Estuarine environments exhibit special ecological characteristics, including intense nutrient inputs, obvious salinity gradients, complex sedimentary dynamics, and periodic wet–dry alternation; together, these environmental stresses shape the structure, function, and ecological adaptability of estuarine microbial communities [2]. Archaea play indispensable roles in key biogeochemical processes in estuarine ecosystems—including ammonia oxidation, methane metabolism, organic matter degradation, and adaptation to extreme conditions—and are therefore important drivers of estuarine ecosystem functional stability [3].

However, despite growing recognition of their importance in biogeochemical cycling, studies of archaeal biogeographic characteristics in estuarine ecosystems remain comparatively limited relative to those of bacteria and fungi [4]. Existing studies have largely focused on describing the basic taxonomic composition of archaeal communities, while the understanding of their distribution patterns across larger spatial scales remained limited [5]. In particular, the ecological responses of microorganisms to macro-environmental gradients (e.g., latitudinal gradients) remain insufficiently resolved [6]. This knowledge gap substantially constrains a comprehensive understanding of archaeal ecological functions in estuarine ecosystems and, consequently, limits the accurate development of microbially driven biogeochemical cycling models [7]. Latitudinal gradients are regarded as one of the primary macro-environmental drivers of global biogeographic patterns, integrating systematic shifts in multiple covarying factors such as temperature, precipitation, solar radiation, seasonality, and vegetation type [8]. In estuarine ecosystems, latitudinal gradients not only directly influence the thermal regimes of the water column and sediments but also indirectly reshape a suite of local environmental conditions—including nutrient concentrations, salinity, dissolved oxygen, redox potential, and sediment physicochemical properties—by modulating watershed climate, riverine inputs, tidal forcing, and the intensity of human activities [9]. The concerted variation in these factors may profoundly influence archaeal community spatial distributions, diversity maintenance, and functional expression by altering microbial niche conditions, resource availability, physiological tolerance ranges, and dispersal capacity [10].

Recent advances in microbial ecological theory indicate that microbial community assembly processes and maintenance are jointly governed by deterministic and stochastic processes [11]. Deterministic processes primarily encompass environmental filtering and niche differentiation, highlighting selection imposed by environmental conditions on microbial survival and reproduction, thereby favoring the persistence and enrichment of taxa with specific functional traits in suitable habitats [12,13]. Stochastic processes, in contrast, include dispersal limitation, ecological drift, mutation, and genetic recombination, underscoring the importance of neutral or near-neutral processes in community assembly and positing that species distributions and abundances are, to a large extent, shaped by random events [14]. In estuarine ecosystems, obvious latitudinal environmental heterogeneity, hydrodynamic regimes, and the degree of geographic isolation may drive systematic shifts in the relative contributions of deterministic versus stochastic processes across latitude bands [15]. However, evidence for how archaeal community assembly mechanisms vary along latitudinal gradients remains scarce, and the observational studies are still lacking on how deterministic and stochastic processes interact under contrasting latitudinal environments to shape the spatial distribution of archaeal communities.

Moreover, microbial ecosystem functioning depends not only on the physiological attributes of individual taxa but also critically on the structure of interspecific interaction networks [16]. Molecular ecological network (MEN) analysis, by resolving correlation patterns among microbial abundances, provides a powerful tool for inferring potential interspecific relationships, including cooperation, competition, predation, and metabolic interactions [17,18]. Topological features of these networks—such as connectivity, clustering coefficient, average path length, and modularity—not only capture the intensity and complexity of within-community interactions but also directly influence community diversity, functional redundancy, and resistance and resilience to environmental perturbations [19,20]. In estuarine archaeal communities, it remains unclear whether archaeal interaction networks vary systematically along latitudinal gradients, whether network complexity and modularity shift with changing environmental conditions, and which environmental factors act as primary drivers of network structural variation. Addressing these questions is essential for clarifying the ecological roles of archaea in estuarine ecosystems and for improving predictions of microbial community responses under climate-change scenarios [21].

China’s coast spans multiple climatic zones—from the temperate Bohai Bay to the tropical South China Sea—forming a pronounced latitudinal gradient that provides an ideal natural laboratory for examining archaeal community responses to macro-environmental change [22]. Here, we selected three representative estuarine regions along the Chinese coastline to systematically investigate latitudinal variation in archaeal community distribution, assembly mechanisms, and interaction network characteristics. Based on ecological theory and previous biogeographic studies, we formulated the following hypotheses: (1) Archaeal species richness varies systematically along the latitudinal gradient, with lower-latitude regions exhibiting higher richness due to greater environmental heterogeneity and resource availability. (2) Community assembly shifts along latitude, with stronger environmental filtering dominating at higher latitudes, whereas stochastic processes (e.g., dispersal limitation and ecological drift) become increasingly important at lower latitudes. (3) Archaeal interaction networks at lower latitudes exhibit greater complexity and modularity, reflecting more intricate ecological interactions and enhanced functional stability. This study aims to develop an integrative ecological framework to reveal how latitudinal gradients regulate archaeal ecological processes and interspecific interactions. In addition, we further validate the effects of key environmental drivers on archaeal communities through controlled laboratory microcosm experiments, thereby disentangling the complex mechanisms underpinning archaeal ecological patterns. Our results provide new microbial evidence for understanding microbially mediated carbon and nitrogen cycling in estuarine ecosystems, assessing mechanisms underlying ecosystem stability and functional maintenance, and improving theoretical predictions of estuarine ecosystem responses under global climate change scenarios.

## 2. Materials and Methods

### 2.1. Study Area and Sampling Design

We selected three representative estuarine regions along the Chinese coastline: the northern region (North China Sea, NCS; 35° N–40° N), the eastern region (East China Sea, ECS; 28° N–32° N), and the southern region (South China Sea, SCS; 18° N–22° N) [23,24]. These regions span distinct climatic zones and encompass multiple covarying environmental gradients—including temperature, salinity, and nutrient concentrations—thereby providing an ideal natural laboratory for investigating latitudinally driven ecological responses of archaeal communities [25]. Sampling was conducted in March 2019 during the high-flow (wet-season) period of the upstream river. This study set up sampling points in 9 intertidal wetlands along the coast of China, located at the estuary areas of the following major rivers: Liaohe River (LH), Haihe River (HH), Yellow River (YR), Sheyanghe River (SYH), Changjiang River (CJ), Oujiang River (OJ), Jiulongjiang River (JLJ), Zhujiang River (ZJ), and Beibuwan Gulf (BBW). In each region, 2–5 sampling stations were established based on spatial heterogeneity across the intertidal zone, with inter-station spacing of 1–3 km to ensure coverage of the major sediment types present in each area. At each station, surface sediments (0–10 cm) were collected using a pre-sterilized stainless-steel corer. Three replicate samples were obtained per station, homogenized, and aliquoted into sterile centrifuge tubes. One subsample was immediately flash-frozen in liquid nitrogen for DNA extraction, whereas the remaining material was sealed and stored at 4 °C for subsequent physicochemical analyses.

### 2.2. Measurement of Environmental Physicochemical Parameters

We quantified multiple physicochemical parameters of sediments and pore water. All samples were air-dried, passed through a 2 mm sieve, and cleared of visible root material before analysis. The measurement of physicochemical parameters in this study followed the same protocols as those used in our previous work [26]. Briefly, dissolved oxygen (DO) values were measured in the overlying water column at the time of sampling. The sediment temperature was measured in situ, and sediment pH was determined using a pH meter (Mettler Toledo, Columbus, OH, USA) after mixing samples with deionized water at a ratio of 1:2.5 (*w*/*v*). Water content was calculated from mass loss after oven-drying at 60 °C to a constant weight, and sediment salinity was measured using a salinometer (YSI Model 30, Xylem Analytics, Yellow Springs, OH, USA). Nitrogen and phosphorus nutrients were analyzed using standard methods. Inorganic nitrogen species (NH_4_^+^-N, NO_2_^−^-N, and NO_3_^−^-N) were extracted with 2 M KCl and quantified using a continuous-flow analyzer (SAN Plus system, Skalar, Breda, The Netherlands). Total phosphorus (TP) was determined by the molybdenum–antimony colorimetric method using a spectrophotometer (UV-5100B, Yuanxi Instrument Co., Ltd., Shanghai, China) following extraction or digestion. Soil organic carbon (SOC) was quantified using an elemental analyzer (Vario Micro Cube, Elementar Trading Co., Ltd., Shanghai, China), whereas sediment total organic carbon (TOC) was determined by the potassium dichromate titration method. Ferrous and ferric iron (Fe^2+^ and Fe^3+^) in sediments were measured using the o-phenanthroline method, while sulfide (S) concentrations were determined by the methylene blue spectrophotometric method.

### 2.3. Microcosm Experiments

Experimental samples were collected in October 2023 from the surface sediments (0–5 cm) of the Yangtze River estuary, where in situ salinity and pH were 15.0 ppt and 7.2, respectively. Anaerobic conditions were established to simulate the prevailing redox environment within estuarine sediments, where oxygen penetration is typically limited despite variable DO levels in overlying waters [27]. After passing through a 2 mm sieve to remove coarse debris, samples were stored at 4 °C in the dark. A three-factor, three-level orthogonal experimental design was employed, incorporating temperature, Fe^3+^, and NO_2_^−^ as environmental factors, with the gradient levels for each factor defined as follows: (1) Temperature: 10, 20, and 30 °C, representing thermal conditions characteristic of high-, mid-, and low-latitude estuaries, respectively. (2) Fe^3+^ concentration: 0.2, 0.5, and 0.8 mg g^−1^ (added as FeCl_3_), simulating variations in ferric iron availability across high-, mid-, and low-latitude estuaries. (3) NO_2_^−^ concentration: 0.5, 0.75, and 1.0 μg g^−1^ (added as NaNO_2_), simulating the heterogeneity of nitrite concentrations in sediments across high-, mid-, and low-latitude estuaries. A total of 27 treatment combinations were established, each with three replicates, along with three control groups receiving only the basal medium without Fe^3+^ or NO_2_^−^ amendments. Each microcosm had a total volume of 50 mL, consisting of 5 g (wet weight) of sediment and 45 mL of artificial seawater medium. The artificial seawater medium was adjusted to a salinity of 30‰ and a pH of 7.2, and its composition followed the Specification for Oceanographic Survey (GB 17378–2007) [28]. Before incubation, the culture systems were purged with high-purity N_2_ (99.999%) for 30 min to remove dissolved oxygen and were then sealed in 120 mL serum bottles. Microcosms were incubated statically in the dark at a constant temperature for 42 d. Following incubation, archaeal 16S rRNA gene copy numbers were quantified by quantitative PCR to assess the potential effects of key environmental factors on archaeal communities.

### 2.4. DNA Extraction, PCR Amplification, qPCR, and High-Throughput Sequencing

Sediment DNA was extracted using the PowerSoil DNA Isolation Kit (MoBio Laboratories, Carlsbad, CA, USA) following the manufacturer’s instructions, with two independent extractions performed for each sample to ensure DNA integrity and reproducibility. The quality of extracted DNA was assessed by electrophoresis on 1% agarose gels, and DNA concentration and purity (A260/A280) were determined using a NanoDrop 2000 spectrophotometer (Thermo Fisher, Shanghai, China). Archaeal community composition was characterized by amplifying the V4–V5 region of the 16S rRNA gene. PCR amplification was performed using the primer pair Arch519F (5′-CAGCCGCCGCGGTAA-3′) and Arch915R (5′-GTGCTCCCCCGCCAATTCCT-3′). The PCR reaction mixture contained 1× high-fidelity buffer, 2.5 mM Mg^2+^, 0.2 mM dNTPs, 0.4 μM of each primer, 1 U of high-fidelity DNA polymerase (Takara, Kusatsu, Shiga, Japan), and approximately 20 ng of template DNA. Thermal cycling conditions were as follows: initial denaturation at 95 °C for 3 min; 30 cycles of denaturation at 95 °C for 30 s, annealing at 55 °C for 30 s, and extension at 72 °C for 45 s; followed by a final extension at 72 °C for 5 min. Purified PCR amplicons were obtained using AMPure XP magnetic beads, pooled to construct sequencing libraries, and subjected to paired-end sequencing (2 × 300 bp) on the Illumina MiSeq platform. All sequencing procedures were performed by Shanghai Majorbio Bio-Pharm Technology Co., Ltd. (Shanghai, China) [29]. Quantitative PCR (qPCR) was used to quantify archaeal 16S rRNA gene copy numbers in samples from the microcosm experiments. The same primer pair, Arch344F (5′-ACGGGGYGCAGCAGGCGCGA-3′) and Arch806R (5′-GGACTACVSGGGTATCTAAT-3′), was used for qPCR amplification. Each qPCR reaction (20 μL) contained 10 μL of SYBR Premix Ex Taq II (Takara, Japan), 0.4 μL of each primer (10 μmol L^−1^), 2 μL of template DNA, and nuclease-free water to a final volume of 20 μL. The qPCR cycling program consisted of an initial denaturation at 95 °C for 5 min, followed by 35 cycles of denaturation at 95 °C for 30 s, annealing at 55 °C for 30 s, and extension at 72 °C for 45 s. Standard curves were generated using 10-fold serial dilutions of plasmids (pMD19-T vector) containing the target gene fragment, spanning concentrations from 10^5^ to 10^9^ copies μL^−1^, with correlation coefficients (R^2^) > 0.99 and amplification efficiencies between 90% and 110%. All samples were analyzed in triplicate, and results are reported as gene copy numbers per gram of dry sediment.

### 2.5. Sequence Processing and Statistical Analyses

Paired-end reads were first assembled using FLASH (V1.2.7, http://ccb.jhu.edu/software/FLASH/), followed by quality filtering according to published standards. Acquired sequences were identified and eliminated using USEARCH based on the UCHIME algorithm, after which sequences were assigned to their respective samples according to barcode information. Sequence clustering was performed with the UPARSE pipeline using the UPARSE-OTU algorithm, and sequences sharing ≥97% similarity were grouped into the same operational taxonomic unit (OTU). Representative sequences from each OTU were taxonomically classified with the RDP classifier. Sequencing depth adequacy was evaluated using rarefaction curves to ensure the robustness of subsequent analyses.

All statistical analyses were performed in the R environment. The alpha-diversity index, including richness indices (ACE and Chao1) and diversity indices (Shannon and Simpson), was calculated using the vegan package in R. Additionally, to further examine latitudinal variation in archaeal α-diversity, we calculated the coefficients of variation (CVs) of diversity indices among samples within each estuarine region, thereby quantifying the degree of within-region dispersion in community diversity. β-diversity was assessed based on Bray–Curtis dissimilarities, visualized using principal coordinates analysis (PCoA), and tested for significant differences among regions using ANOSIM [30]. To assess similarities in archaeal community composition among samples from different estuarine regions, hierarchical clustering analysis was performed based on the relative abundances of the top 100 most abundant OTUs. And the hierarchical clustering was conducted using the unweighted pair group method with arithmetic mean (UPGMA). To identify key environmental drivers of archaeal community structure, redundancy analysis (RDA) was applied to examine the linear relationships between community composition and environmental variables. Mantel tests were performed in R to evaluate the correlation between pairwise distance matrices, assessing the relationships between community structure and environmental factors. All environmental variables were standardized before analysis to minimize biases arising from differences in scale. The neutral community model (NCM) was applied to assess the contributions of random processes and environmental filtering to the archara community assembly. The fit of the model was determined by calculating the goodness-of-fit values (R) and migration rates (M) for archara communities using the micropower and NCM packages. Ecological assembly processes were inferred using the beta Nearest Taxon Index (βNTI) in combination with the Raup–Crick metric based on Bray–Curtis dissimilarity (RC_bray_) [31]. Values of βNTI > +2 or <−2 indicate that phylogenetic turnover is dominated by deterministic processes (environmental filtering or niche differentiation). When −2 < βNTI < +2, stochastic processes—including dispersal limitation, homogenizing dispersal, or ecological drift—were distinguished using (RC_bray_). This approach enabled quantitative evaluation of differences in the relative importance of ecological processes across latitudinal regions. Co-occurrence networks were constructed using the SparCC algorithm—which accounts for data sparsity and compositionality in microbial relative-abundance data—by retaining associations with absolute correlation coefficients |r| > 0.6 and statistical significance at *p* < 0.05 [32]. Network visualization and computation of topological parameters—including degree centrality, clustering coefficient, average path length, and modularity—were performed using Gephi (v0.9.2) [33,34]. Network stability was analyzed via co-occurrence networks using robustness simulations and vulnerability metrics, including vulnerability indices, as well as the proportion of negative correlations. These indices were used to compare the stability and resistance of archaeal community networks across regions.

## 3. Results

### 3.1. Latitudinal Patterns of Environmental Variables and Archaeal Community Diversity

**Figure 1:**
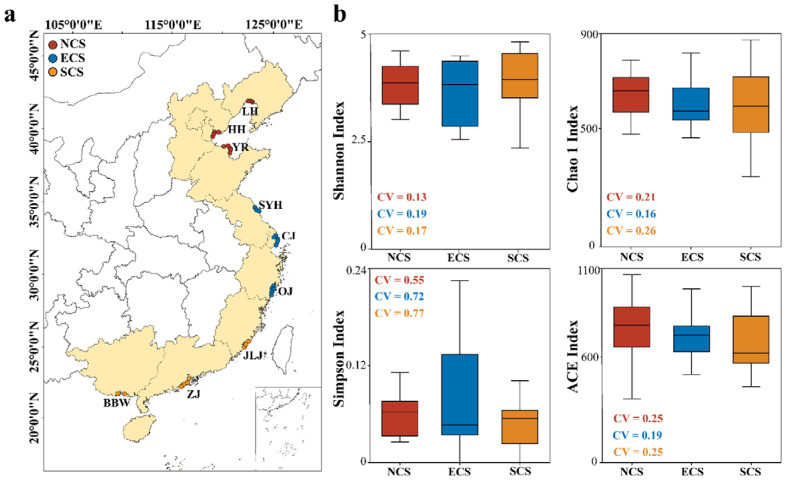
**Sampling sites and the diversity characteristics of archaeal communities across different estuarine regions.** (**a**) The 9 sampling areas selected for this study are LH, Liaohe River; HH, Haihe River; YR, Yellow River; SYH, Sheyanghe River; CJ, Changjiang River; OJ, Oujiang River; JLJ, Jiulongjiang River; ZJ, Zhujiang River; BBW, Beibuwan Gulf. (**b**) Boxplots illustrate the α-diversity indices within each estuarine region, including the NCS (North China Sea estuaries, 35–40° N), ECS (East China Sea estuaries, 28–32° N), and SCS (South China Sea estuaries, 18–22° N). The line within each box denotes the median, the box boundaries represent the 25th and 75th percentiles, and the whiskers extend to the minimum and maximum values. CV refers to the coefficient of variation; higher CV values indicate greater spatial heterogeneity of archaeal diversity within a given estuarine region.

The physicochemical characteristics of sediments at all sampling sites are summarized in Table 1. Except for pH and sulfide, sediment physicochemical parameters differed significantly among the three estuarine regions (two-way ANOVA, *p* < 0.05). Specifically, sediment temperature exhibited a clear south-to-north decreasing trend, with mean values of 21.27 °C, 20.66 °C, and 15.04 °C in the SCS, ECS, and NCS regions, respectively. The salinity showed the opposite pattern, being significantly higher in the NCS (mean = 22.19) than in the ECS (mean = 5.84) and SCS (mean = 8.02). Additionally, sediments from the NCS estuarine region exhibited significantly higher concentrations of DO, NO_2_^−^, NO_3_^−^, NH_4_^+^, and Fe^2+^, whereas SOC, MC, and Fe^3+^ concentrations were significantly lower. Although the differences were not statistically significant, the mean values of the ACE and Chao1 indices across the three latitudinal regions generally decreased as the latitudes decreased. Specifically, mean ACE values were 758.18, 672.14, and 644.36 for the NCS, ECS, and SCS regions, respectively, while mean Chao1 values were 629.66, 565.30, and 573.36, respectively. In terms of species diversity, the Shannon index was highest in the SCS region (range 0 to 4.81), clearly exceeding values observed in the other two regions. In contrast, the Simpson index reached its highest values in the ECS region (range 0 to 0.22), suggesting that communities in this region may be dominated by a limited number of taxa (Figure 1). The within-region CV of the Shannon index was 0.13 in the NCS, 0.19 in the ECS, and 0.17 in the SCS. In contrast, the CV of the Simpson index exhibited an increasing trend from the NCS to the SCS, with values of 0.55, 0.72, and 0.77, respectively. The CV values of the ACE and Chao1 indices were lowest in the ECS region, indicating a relatively stable distribution of species richness. This pattern might be associated with the transitional nature of mid-latitude estuarine ecosystems, where moderate environmental conditions promote balanced species composition and reduced variability.

### 3.2. Spatial Separation of Archaeal Communities Along the Latitudinal Gradient and the Influence of Environmental Variables

**Figure 2:**
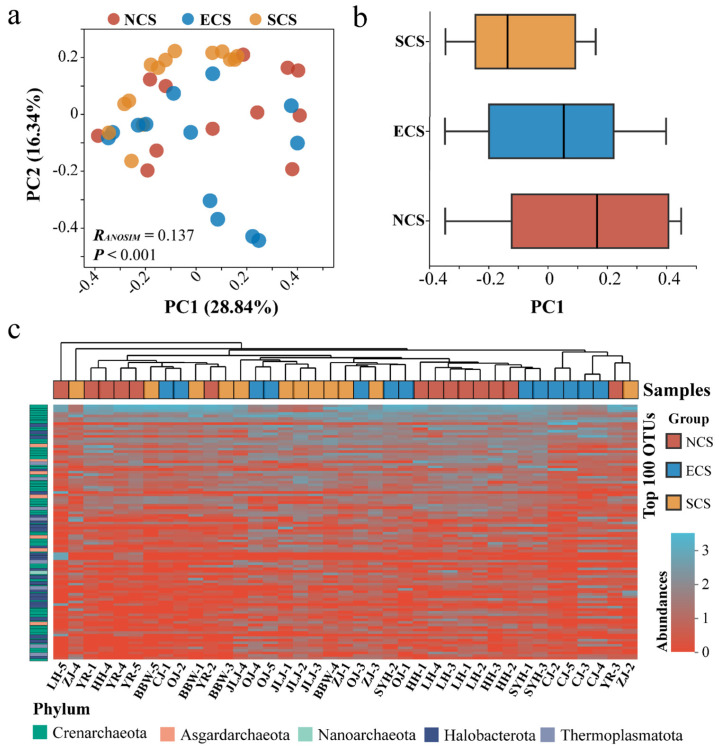
**Spatial differentiation of archaeal communities among estuarine regions along the latitudinal gradient.** (**a**) Principal coordinates analysis (PCoA) based on Bray–Curtis dissimilarities illustrating the spatial distribution of archaeal communities. Samples from different regions are indicated by distinct colors: NCS (red circles), ECS (blue circles), and SCS (yellow circles). The first two axes explain 28.84% and 16.34% of the total variation, respectively. (**b**) Boxplots showing sample scores along PCoA axis 1 for the three estuarine regions. (**c**) Hierarchical clustering dendrogram based on the abundance of the top 100 OTUs (after log10-transformed). The color gradients of the rows and columns of the heatmap, respectively, represent the sampling groups and the phylum of the top 100 OTUs.

**Figure 3:**
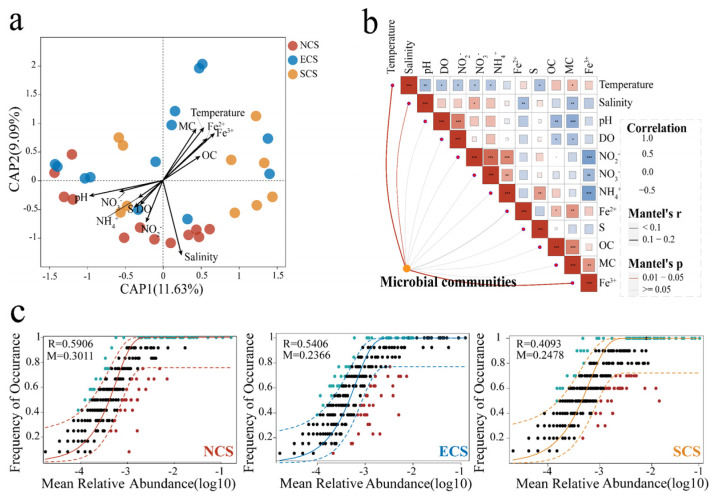
**Relationships between archaeal communities and environmental variables, and characteristics of NCM results across estuarine regions.** (**a**) RDA ordination showing the relationships between archaeal community composition and key environmental variables. Samples are color-coded by region (NCS, red; ECS, blue; SCS, yellow). Arrow length indicates each variable’s relative explanatory power. (**b**) Mantel test heatmap summarizing correlation strength (Mantel’s r) and significance between environmental variables and archaeal community structure. The width and color of the lines, respectively, indicate Mantel’s r and Mantel’s *p* values. (**c**) NCM analysis showing the migration rate (M) and model goodness-of-fit (R) across regions, the asterisks indicate the statistical significance of the correlation (* *p* < 0.05, ** *p* < 0.01, *** *p* < 0.001).

PCoA based on Bray–Curtis dissimilarities showed that samples from the NCS, ECS, and SCS regions were not clearly separated in the two-dimensional ordination space. Nevertheless, ANOSIM results indicated that the β-diversity of archaeal community separation was significantly different among estuarine regions (R = 0.137, *p* < 0.001) (Figure 2a). In addition, boxplots of sample scores along PCoA axis 1 revealed a clear gradient from low to high latitudes across the three estuarine regions, further indicating the spatial differentiation of archaeal communities along the latitudinal gradient (Figure 2b). Furthermore, hierarchical clustering based on the top 100 most abundant OTUs showed that samples from the same estuarine region tended to cluster together, suggesting that inter-regional differences in environmental conditions exert a potential influence on archaeal biogeographic distributions (Figure 2c). The results of RDA indicated that temperature, salinity, pH, NO_2_^−^, Fe^3+^, Fe^2+^, and MC significantly structured the spatial heterogeneity of archaeal communities, jointly explaining approximately 83.42% of the total community variation (Figure 3a). Consistently, Mantel tests identified temperature, salinity, and Fe^3+^ as key drivers of archaeal community patterns, further underscoring the regulatory role of critical sediment environmental variables (Figure 3b). The NCM analyses showed that the R values for the NCS, ECS, and SCS regions were 0.5906, 0.5406, and 0.4093, respectively, with corresponding migration rates (M values) of 0.3011, 0.2366, and 0.2478 (Figure 3c). Comparatively, archaeal communities in the NCS region exhibited the highest R and M values, indicating a stronger influence of stochastic processes and suggesting enhanced dispersal capacity in this region.

### 3.3. Latitudinal Patterns of Archaeal Community Assembly Processes

**Figure 4:**
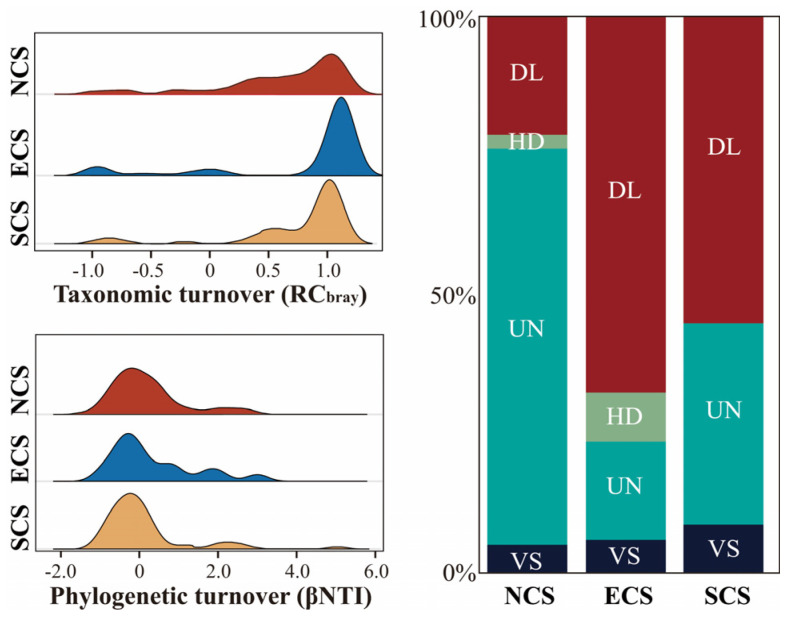
**Distributions of βNTI and RC_bray_ values and inferred community assembly processes of archaeal communities across estuarine regions.** The ridge plots illustrate the distributions and variation patterns of βNTI and RC_bray_ values of the three estuarine regions. Bar charts depict the relative contributions of community assembly processes in the NCS, ECS, and SCS regions. DL, dispersal limitation; VS, variable selection; UN, undominated (stochastic) processes; HD, homogenizing dispersal. NCS, ECS, and SCS represent the estuarine regions of the North China Sea, East China Sea, and South China Sea, respectively.

The βNTI values of archaeal communities in all three estuarine regions were predominantly distributed between −1.0 and 1.0, which indicated that stochastic processes dominated community assembly across regions (with the highest contribution in the NCS with the value of 95.00%, followed by the ECS with the value of 94.12%). In contrast, the SCS exhibited the lowest relative contribution of stochastic processes, accounting for 91.33% of community assembly. In addition, dispersal limitation (DL) and homogenizing dispersal (HD) together accounted for the highest proportion in the mid-latitude region (85.29%), exhibiting a pattern of increasing from north to mid-latitudes and then decreasing toward the south. Notably, the HD process was not detected in the SCS region, where stochastic assembly was primarily dominated by DL and undominated (UN) processes (Figure 4). Overall, our results indicate that environmental filtering plays a more important role at lower latitudes, whereas DL becomes more prominent at mid-latitudes, revealing a clear latitudinal gradient in the relative importance of deterministic and stochastic processes.

### 3.4. Archaeal Communities MEN Characteristics and Stability of Network Along the Latitudinal Gradient

**Figure 5:**
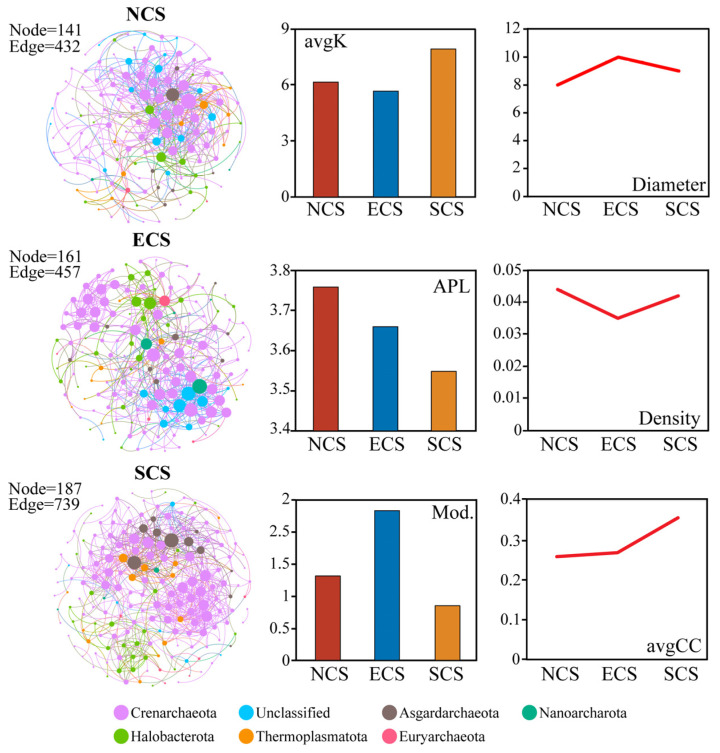
**MEN characteristics of archaeal communities in estuarine regions across China.** NCS, ECS, and SCS represent the northern, eastern, and southern intertidal estuarine regions of China, respectively. Node colors indicate major archaeal phyla, and node size is proportional to node degree. Bar charts and line plots display network metrics for each region (NCS, ECS, and SCS), including average degree (avgK), network diameter, average path length (APL), network density, modularity (Mod.), and average clustering coefficient (avgCC).

From the northern to southern estuaries, obvious spatial differentiation was observed in the molecular ecological network properties of archaeal communities. The NCS, ECS, and SCS MEN comprised 141, 161, and 187 nodes, with 432, 457, and 7739 edges, respectively. Network topology metrics indicated a decreasing trend in average path length (APL) from the NCS to the SCS. In contrast, the average degree (avgK) exhibited a non-monotonic pattern, decreasing initially and then increasing toward lower latitudes; similar trends were observed for network density and the average clustering coefficient (avgCC). Notably, network modularity and network diameter exhibited the opposite pattern, increasing from the NCS to the ECS and then decreasing toward the SCS. These results suggest that archaeal networks in the SCS possess the highest connectivity and structural complexity, whereas those in the ECS display a more compartmentalized architecture with relatively higher modularity (Figure 5). Furthermore, network stability analyses revealed pronounced regional differences. Archaea in the NCS exhibited the weakest network stability, characterized by a steeper robustness decay slope, higher network vulnerability, and a greater proportion of negative correlations (vulnerability index = 0.212; proportion of negative links = 10.000). This was followed by the ECS estuaries (vulnerability index = 0.119; proportion of negative links = 8.050). In contrast, archaeal networks in the SCS showed the highest stability, with the lowest vulnerability index (0.099) and the smallest proportion of negative correlations (6.100). These results suggest that the stability of archaeal community networks is closely linked to their structural complexity and connectivity. The highly connected and closely interacting network observed in the SCS region likely enhances resistance to external environmental disturbances and facilitates recovery following perturbations, thereby sustaining a higher level of ecological stability.

### 3.5. Effects of Key Environmental Factors (Temperature, Fe^3+^, and NO_2_^−^) on Archaeal Abundance

Given the pivotal roles of temperature, Fe^3+^, and NO_2_^−^ in shaping the ecological characteristics of archaeal communities in Chinese estuaries (Figure 3a,b), we further validated their potential effects through controlled laboratory experiments (Table 2). ANOVA analysis revealed that temperature exerted a highly significant effect on archaeal abundance (F = 45.32, *p* < 0.001); Fe^3+^ concentration also had a significant influence (F = 18.67, *p* < 0.01), whereas the effect of NO_2_^−^ was relatively weak and not statistically significant (F = 3.21, *p* > 0.05). Moreover, the temperature effect exhibited a clear gradient: the mean growth fold change at 30 °C (1.12 ± 0.52) was significantly higher than that at 20 °C (0.67 ± 0.30) and 10 °C (0.41 ± 0.17), indicating that elevated temperature markedly stimulated archaeal proliferation. The effect of Fe^3+^ concentration followed a nonlinear pattern, with the intermediate (0.5 mg g^−1^) and high (0.8 mg g^−1^) treatments showing mean growth fold changes of 0.94 and 0.91, respectively—both significantly higher than that of the low-concentration treatment (0.35). However, no significant difference was detected between the 0.5 and 0.8 mg g^−1^ levels, suggesting a saturation threshold in the growth-promoting effect of Fe^3+^. In contrast, NO_2_^−^ exhibited a negative effect on archaeal growth: the low-concentration treatment (0.5 μg g^−1^) showed a significantly higher growth fold change (0.90) than the intermediate (0.75 μg g^−1^, 0.72) and high (1.0 μg g^−1^, 0.58) treatments, indicating that elevated NO_2_^−^ levels may inhibit archaeal proliferation. In addition, a significant temperature × Fe^3+^ interaction was detected (F = 8.94, *p* < 0.01; Table 2). Under the 30 °C condition, the stimulatory effect of Fe^3+^ was markedly enhanced, with the 30 °C + 0.5 mg g^−1^ Fe^3+^ treatment (T3Fe2N1) exhibiting the highest growth fold change (1.85) among all treatments. This result confirms a synergistic effect of temperature and Fe^3+^ on archaeal abundance.

## 4. Discussion

This study systematically demonstrates a multi-level mechanism by which macro-scale latitudinal gradients shape archaeal biogeographic patterns through coordinated effects on local environmental conditions, community assembly processes, and interaction networks. Our results not only reveal the latitudinal spatial separation in archaeal community structure but also demonstrate that assembly mechanisms of archaeal communities in Chinese estuarine sediments undergo a non-monotonic shift along the latitudinal gradient, which differs markedly from our initial hypothesis. Additionally, our null-model analyses reveal a complex scenario of archaral communities: stochastic processes accounted for up to 95% of community assembly in the high-latitude NCS region, a proportion substantially higher than that observed in the low-latitude SCS region. This pattern may stem from the pronounced seasonal variability and frequent disturbance events characteristic of temperate estuarine ecosystems.

### 4.1. Regulation of Archaeal Diversity and Community Assembly Processes Along the Latitudinal Gradient

Our results indicate that, although latitudinal trends in α-diversity indices (e.g., ACE and Chao1) were not statistically significant, results of β-diversity and clustering analyses based on the top 100 OTUs suggest a discernible degree of latitudinal differentiation in archaeal community structure. This finding indicates that changes in archaeal community species structure (compositional turnover) constitute the primary mode of response to the latitudinal gradient. RDA and Mantel tests further linked this differentiation to key environmental drivers—such as temperature, salinity, and Fe^3+^—underscoring the fundamental role of environmental filtering in shaping large-scale biogeographic patterns [35]. Further analyses of archaeal community assembly revealed a more complex pattern of community construction. Our results indicate that stochastic processes—primarily dispersal limitation and ecological drift—accounted for up to 95% of archaeal community assembly in the high-latitude NCS region. This finding may be associated with strong seasonal environmental fluctuations, frequent disturbance events (e.g., storm surges and runoff pulses), and the resulting “niche compression” effects in estuarine systems. In high-latitude regions, pronounced seasonal temperature fluctuations, irregular storm-surge events, and runoff pulses between wet and dry periods collectively create highly dynamic and unpredictable environmental conditions [2]. Under such non-equilibrium conditions, classical deterministic environmental filtering may fail to establish stable dominant taxa, as the rate of environmental change may exceed the capacity of microbial communities to respond and adapt [36]. This interpretation is supported by the observation that network stability was lowest in the NCS region, as highly dynamic community structures are less likely to form stable and tightly coupled interspecific interaction networks [37].

### 4.2. Decoupling of Network Complexity and Stability in Archaeal Communities

Another important finding of this study is that community assembly processes do not exhibit a simple linear correspondence with network topological features but instead display a complex pattern of decoupling. Archaeal networks in the SCS, although shaped predominantly by relatively strong deterministic processes, exhibited the highest levels of connectivity, structural complexity, and stability. In contrast, networks in the NCS were dominated by highly stochastic assembly processes and displayed the sparsest and least stable structural characteristics. This lack of congruence challenges classical ecological theory by calling into question whether greater determinism in community assembly necessarily translates into higher network complexity [4]. One probable explanation is that in the low-latitude SCS region, relatively stable environmental conditions and sustained resource supply may facilitate fine-scale niche differentiation, allowing the coexistence of a greater number of ecologically similar taxa. This “niche complementarity” mechanism not only strengthens deterministic selection but also promotes the formation of more complex metabolic interaction networks through functional trait differentiation. Consequently, the highly connected and tightly organized network structure observed in the SCS region may reflect a mature ecosystem in which intense competition and niche differentiation ultimately give rise to functional complementarity and highly organized interactions, providing renewed support for the “complexity–stability” hypothesis in microbial ecosystems [38]. In contrast, in the high-latitude NCS region, frequent environmental disturbances may impede the establishment of microscopic niches, causing communities to rely more frequently on species turnover and recolonization to maintain ecosystem functioning, rather than on the development of stable, long-term interspecific interactions [39]. Notably, the ECS region exhibited the highest degree of modularity, a pattern that closely aligns with the pronounced contribution of dispersal limitation processes in this region [40]. A modular network can localize ecological dynamics and constrain the propagation of disturbances, potentially representing an adaptive response to moderate environmental heterogeneity and geographic isolation [41]. Networks with higher modularity tend to exhibit greater functional redundancy and resilience, such that even when certain modules are disrupted, other modules can continue to sustain core ecosystem functions. Such network topology may represent a key mechanism by which estuarine ecosystems maintain functional stability under complex hydrodynamic conditions [42].

### 4.3. Ecological Implications of Key Environmental Drivers

Finally, we emphasize the key environmental drivers in this study. In the low-latitude SCS region, higher temperatures may promote anaerobic metabolic networks centered on iron reduction, encompassing iron reduction, methanogenesis, sulfate reduction, and other syntrophic processes. These metabolic pathways often require complex interspecific electron transfer and metabolic cross-feeding networks to operate in a coordinated manner. For example, syntrophic associations based on hydrogen or formate transfer may occur between methanogenic archaea and iron-reducing microorganisms, whereas competitive and cooperative interactions between sulfate-reducing microorganisms and methanogens can directly regulate carbon cycling rates in sediments [43]. Such metabolism-driven interactions are likely to give rise to the highly connected, structurally complex, and well-organized network characteristics observed in SCS archaeal communities. In contrast, sediments in the high-latitude NCS region—characterized by lower temperatures and lower Fe^3+^ concentrations—are likely to remain relatively oxidized or only weakly reducing, with consequently reduced intensity of iron-reduction metabolism. Under such conditions, denitrification, iron reduction, and aerobic respiration may co-occur but at relatively low intensities, without a clearly dominant metabolic pathway in archaeal groups, thereby resulting in looser and more unstable interspecific interactions within the community. Accordingly, although further experiments are needed for verification, we hypothesize that in environments lacking a clearly dominant metabolic pathway, communities may rely more heavily on opportunistic resource utilization and rapid species turnover, rather than on the establishment of stable, long-term metabolic interactions to sustain ecosystem functioning.

### 4.4. Experimental Validation of the Synergistic Effects of Temperature, Fe^3+^, and NO_2_^−^ on Archaeal Community Dynamics

Through controlled laboratory incubation experiments, we systematically validated the core hypotheses derived from field investigations, demonstrating that temperature is the dominant driver of archaeal community dynamics in estuarine sediments. Archaeal abundance increased significantly under the 30 °C treatment (mean increase of 1.12-fold), whereas growth was markedly constrained at 10 °C (mean increase of only 0.41-fold). This study establishes a direct causal relationship between temperature and archaeal abundance, revealing the physiological mechanisms by which elevated temperatures enhance archaeal metabolic activity and proliferation rates. Fe^3+^, acting as an electron acceptor, significantly stimulated archaeal growth within the range of 0.5–0.8 mg g^−1^ and exhibited a clear saturation effect. Moreover, Fe^3+^ interacted synergistically with temperature through a positive feedback mechanism: sufficient Fe^3+^ availability promotes the development of anaerobic iron-reducing metabolic networks, thereby creating favorable microenvironments and metabolic substrates for methanogenic archaea, whereas concentrations exceeding a threshold may induce oxidative damage via Fenton reactions [44]. Contrary to expectations, elevated NO_2_^−^ exerted a pronounced inhibitory effect on archaeal growth (with only a 0.58-fold increase in the high-concentration treatment), revealing competitive interactions between denitrifiers and methanogenic archaea at the level of metabolic pathways. We hypothesize that high NO_2_^−^ availability may constrain archaeal growth through a dual mechanism involving substrate competition and the accumulation of toxic intermediate products [45]. It is worth noting that the microcosm experiments were conducted under controlled conditions that may not fully replicate the complexity of natural estuarine ecosystems. Additionally, the experimental sediments were collected in 2023, and thus, temporal differences from the 2019 field samples may limit direct comparisons. The observed responses should be interpreted as indicative trends rather than precise predictions, with future studies needed to confirm these findings under natural conditions. However, these findings provide new evidence for trade-offs among distinct anaerobic metabolic pathways in estuarine ecosystems. Based on our incubation system, we predict that a future temperature increase of 2–4 °C could lead to a 20–40% increase in archaeal abundance, potentially enhancing methanogenic activity and methane emissions, while shifts in nitrogen loading may further regulate carbon cycling pathways by altering metabolic competition.

## 5. Conclusions

In summary, this study provides an integrative ecological framework that elucidates the complex mechanisms by which macro-environmental gradients shape microbial biogeographic patterns through multiple, interconnected pathways, including environmental filtering, dispersal dynamics, and archaeal interactions. Our findings underscore that predicting the impacts of global change (e.g., climate warming) on estuarine microbial communities requires attention not only to shifts in species composition but also to the reorganization of community assembly processes and co-occurrence network structures, which may exert more profound effects on ecosystem functional stability. Overall, our study provides an essential microbial ecological basis for assessing ecological responses of estuarine ecosystems to climate change and for informing the development of adaptive management strategies. However, this study also has several limitations. First, MEN inferred from correlation analyses cannot discriminate between direct interactions and indirect associations, nor can they explicitly resolve the nature of relationships such as mutualism versus competition. Second, single-time-point sampling is insufficient to capture the influence of seasonal dynamics on community assembly processes and network stability, and our sampling period may not fully represent ecological processes occurring across different seasons. Third, 16S rRNA gene-based data have inherent limitations for functional inference, as they cannot be directly linked to specific metabolic pathways or rates of biogeochemical processes. Fourth, this study primarily infers the potential regulatory effects of key environmental factors based on archaeal abundance patterns and thus lacks more detailed evidence to precisely characterize their mechanistic influences.

Based on these considerations, we propose the following directions for future research: (1) Integrate metagenomic, meta-transcriptomic, and metaproteomic approaches to directly resolve key metabolic pathways and the functional basis of cross-domain interactions. (2) Conduct multi-seasonal and multi-year time-series observations to elucidate the temporal dynamics of community assembly processes and network structures. (3) Integrate in situ stable isotope tracing with more finely controlled microcosm manipulation experiments to verify the causal effects of key environmental factors (e.g., Fe^3+^ and dissolved oxygen) on the structure and stability of archaeal community networks. (4) Quantitatively link cross-domain archaeal–bacterial–fungal interaction networks with rates of specific biogeochemical processes (e.g., methane fluxes, nitrification rates, and denitrification efficiency) to develop predictive models that scale from microscopic interactions to macroscopic ecosystem functions.

## Figures and Tables

**Table 1:** Sediment characteristics of sampling sites from NCS to SCS.

Sample ID	Temperature	Salinity	pH	DO	NO_2_^−^	NO_3_^−^	NH_4_^+^	Fe^2−^	S	OC	MC	Fe^3−^	Group
	(°C)	(ppt)		%	(μg/g)	(μg/g)	(μg/g)	(mg/g)	(μg/g)	(mg/g)	%	(mg/g)	
LH-1	14.30	27.00	8.15	18.40	0.52	11.47	49.40	0.69	0.03	9.53	28.00	0.20	NCS
LH-2	12.00	26.80	7.93	18.20	0.61	9.71	53.77	1.05	0.01	16.26	40.59	0.35	NCS
LH-3	11.00	23.10	8.31	22.10	0.46	8.87	20.54	0.63	0.29	8.69	27.03	0.39	NCS
HH-1	15.40	28.20	7.42	4.10	0.74	11.49	44.74	0.99	0.04	17.11	51.59	0.26	NCS
HH-2	22.20	26.00	7.73	5.90	0.53	8.43	42.63	0.60	0.01	7.14	26.00	0.26	NCS
HH-3	14.70	25.60	8.16	7.50	0.48	7.27	23.52	1.30	1.70	13.36	28.61	0.39	NCS
HH-4	16.20	25.60	8.07	18.12	0.38	7.49	31.93	0.42	0.01	8.66	25.06	0.61	NCS
YR-1	11.10	27.40	7.76	6.60	0.41	8.15	26.29	0.35	0.13	7.14	27.59	0.41	NCS
YR-2	13.30	24.30	7.82	21.90	0.34	8.05	33.95	0.67	1.50	12.24	30.99	0.27	NCS
YR-3	15.10	0.56	8.63	29.10	0.44	9.78	30.10	0.33	0.06	6.33	31.81	0.45	NCS
YR-4	16.50	29.00	8.44	7.60	0.24	7.99	11.45	0.06	0.01	3.49	21.82	0.36	NCS
YR-5	14.20	7.18	8.67	8.00	0.47	7.82	31.81	0.36	0.04	5.97	25.24	0.27	NCS
SYH-1	16.00	22.90	7.99	5.50	0.46	7.14	24.32	0.69	0.05	6.77	30.27	0.31	NCS
SYH-2	16.70	16.60	8.04	6.50	0.31	8.52	22.27	0.55	0.02	5.19	37.19	0.48	NCS
SYH-3	16.90	22.60	7.99	32.80	0.20	7.69	11.12	0.51	0.04	3.83	26.81	0.46	NCS
CJ-1	23.80	0.16	7.66	6.90	0.37	8.81	42.62	1.34	0.01	10.81	39.42	0.33	ECS
CJ-2	21.20	0.22	7.01	6.70	0.51	9.04	112.05	1.52	0.01	11.81	40.95	0.19	ECS
CJ-3	26.50	2.42	7.94	7.10	0.24	8.85	14.42	1.13	0.01	12.00	39.13	0.68	ECS
CJ-4	26.50	2.42	7.94	7.10	0.28	7.54	14.60	1.11	0.01	22.41	49.41	0.80	ECS
CJ-5	25.10	10.95	7.92	9.00	0.26	8.12	12.54	0.81	0.01	7.15	37.48	0.64	ECS
OJ-1	14.10	7.64	7.91	15.50	0.19	2.24	12.49	2.10	0.02	6.74	40.74	0.84	ECS
OJ-2	18.70	1.75	8.60	15.50	0.15	3.07	6.94	2.05	0.00	8.33	45.33	0.82	ECS
OJ-3	14.30	2.16	7.52	1.30	0.20	3.52	11.09	1.92	0.00	12.10	40.89	1.03	ECS
OJ-4	16.90	13.20	7.63	15.10	0.17	4.07	7.62	1.98	0.01	10.11	42.96	1.21	ECS
OJ-5	19.50	17.50	7.63	14.00	0.17	1.97	9.75	1.49	0.00	8.13	36.04	0.81	ECS
JLJ-1	19.40	19.10	7.12	2.10	0.23	2.88	25.22	0.73	0.05	7.09	26.39	0.22	SCS
JLJ-2	22.20	14.90	7.38	1.40	0.25	2.59	8.45	0.74	0.01	13.24	49.35	1.40	SCS
JLJ-3	25.30	19.50	7.57	1.50	0.24	2.29	13.21	0.68	0.01	11.12	48.42	1.30	SCS
JLJ-4	25.10	6.14	7.82	4.40	0.27	1.38	16.05	1.46	0.00	18.38	36.29	0.23	SCS
ZJ-1	19.50	3.16	7.57	3.00	0.20	4.28	20.35	0.47	0.01	16.21	42.05	1.70	SCS
ZJ-2	17.80	1.16	7.42	1.00	0.26	4.02	34.18	1.14	0.50	19.20	49.93	0.76	SCS
ZJ-3	18.80	5.47	7.08	1.20	0.23	4.78	26.90	1.30	0.06	16.28	53.54	1.34	SCS
ZJ-4	18.30	0.49	7.67	1.40	0.22	3.37	20.52	2.69	0.04	9.52	34.63	1.05	SCS
BBW-1	21.70	1.75	7.76	8.90	0.09	0.44	101.56	0.64	0.49	15.88	31.21	0.32	SCS
BBW-2	24.60	8.54	7.57	9.00	0.08	1.36	28.76	0.12	0.21	13.00	38.09	0.70	SCS

LH, Liaohe River; HH, Haihe River; YR, Yellow River; SYH, Sheyanghe River; CJ, Changjiang River; OJ, Oujiang River; JLJ, Jiulongjiang River; ZJ, Zhujiang River; BBW, Beibuwan Gulf. NCS, Northern Coastal Seas; ECS, East China Sea; SCS, South China Sea. The prefix letters in the sample numbers (such as LH-1, CJ-2 in Table 1) correspond to the abbreviations of the above locations, and the numbers represent the sampling points in the same coastal location.

**Table 2:** The combination of controlled laboratory experiments and effects on archaeal richness.

Experiment Number	Temperature (°C)	Fe^3+^ (mg/g)	NO_2_^−^ (μg/g)	Pre-Culture Abundances (Copies/g)	Post-Culture Abundances (Copies/g)	Multiple Growth
T1Fe1N1	10.00	0.20	0.50	1.57 × 10^7^	4.28 × 10^6^	0.27
T1Fe1N2	10.00	0.20	0.75	1.48 × 10^7^	3.04 × 10^6^	0.21
T1Fe1N3	10.00	0.20	1.00	1.60 × 10^7^	2.62 × 10^6^	0.16
T1Fe2N1	10.00	0.50	0.50	1.73 × 10^7^	1.01 × 10^7^	0.59
T1Fe2N2	10.00	0.50	0.75	1.46 × 10^7^	8.48 × 10^6^	0.58
T1Fe2N3	10.00	0.50	1.00	1.46 × 10^7^	5.74 × 10^6^	0.39
T1Fe3N1	10.00	0.80	0.50	1.74 × 10^7^	9.79 × 10^6^	0.56
T1Fe3N2	10.00	0.80	0.75	1.62 × 10^7^	8.68 × 10^6^	0.54
T1Fe3N3	10.00	0.80	1.00	1.43 × 10^7^	5.06 × 10^6^	0.35
T2Fe1N1	20.00	0.20	0.50	1.58 × 10^7^	7.07 × 10^6^	0.45
T2Fe1N2	20.00	0.20	0.75	1.43 × 10^7^	4.34 × 10^6^	0.30
T2Fe1N3	20.00	0.20	1.00	1.43 × 10^7^	3.58 × 10^6^	0.25
T2Fe2N1	20.00	0.50	0.50	1.54 × 10^7^	1.60 × 10^7^	1.04
T2Fe2N2	20.00	0.50	0.75	1.21 × 10^7^	1.08 × 10^7^	0.89
T2Fe2N3	20.00	0.50	1.00	1.24 × 10^7^	8.22 × 10^6^	0.66
T2Fe3N1	20.00	0.80	0.50	1.42 × 10^7^	1.44 × 10^7^	1.02
T2Fe3N2	20.00	0.80	0.75	1.35 × 10^7^	1.11 × 10^7^	0.82
T2Fe3N3	20.00	0.80	1.00	1.55 × 10^7^	8.91 × 10^6^	0.58
T3Fe1N1	30.00	0.20	0.50	1.36 × 10^7^	7.92 × 10^6^	0.58
T3Fe1N2	30.00	0.20	0.75	1.29 × 10^7^	6.25 × 10^6^	0.49
T3Fe1N3	30.00	0.20	1.00	1.72 × 10^7^	7.31 × 10^6^	0.43
T3Fe2N1	30.00	0.50	0.50	1.47 × 10^7^	2.71 × 10^7^	1.85
T3Fe2N2	30.00	0.50	0.75	1.51 × 10^7^	1.91 × 10^7^	1.26
T3Fe2N3	30.00	0.50	1.00	1.29 × 10^7^	1.51 × 10^7^	1.17
T3Fe3N1	30.00	0.80	0.50	1.42 × 10^7^	2.47 × 10^7^	1.74
T3Fe3N2	30.00	0.80	0.75	1.52 × 10^7^	2.11 × 10^7^	1.39
T3Fe3N3	30.00	0.80	1.00	1.33 × 10^7^	1.59 × 10^7^	1.20

## Data Availability

Sequence data were deposited in the National Center for Biotechnology Information (NCBI) Sequence Read Archive with BioProject PRJNA755846. Other data are available from the corresponding author upon reasonable request.
